# Synthetic MRI plus FSE-PROPELLER DWI for differentiating malignant from benign head and neck tumors: a preliminary study

**DOI:** 10.3389/fonc.2023.1225420

**Published:** 2023-09-27

**Authors:** Baohong Wen, Zanxia Zhang, Jing Zhu, Liang Liu, Zijun Liu, Xiaoyue Ma, Kaiyu Wang, Lizhi Xie, Yong Zhang, Jingliang Cheng

**Affiliations:** ^1^Department of MRI, the First Affiliated Hospital of Zhengzhou University, Zhengzhou, China; ^2^MR Research China, GE Healthcare, Beijing, China

**Keywords:** synthetic, magnetic resonance imaging, diffusion-weighted image, apparent diffusion coefficient, head and neck tumors

## Abstract

**Background:**

Preoperative classification of head and neck (HN) tumors remains challenging, especially distinguishing early cancerogenic masses from benign lesions. Synthetic MRI offers a new way for quantitative analysis of tumors. The present study investigated the application of synthetic MRI and stimulus and fast spin echo diffusion-weighted imaging with periodically rotated overlapping parallel lines with enhanced reconstruction (FSE-PROPELLER DWI) to differentiate malignant from benign HN tumors.

**Materials and methods:**

Forty-eight patients with pathologically confirmed HN tumors were retrospectively recruited between August 2022 and October 2022. The patients were divided into malignant (n = 28) and benign (n = 20) groups. All patients were scanned using synthetic MRI and FSE-PROPELLER DWI. T1, T2, and proton density (PD) values were acquired on the synthetic MRI and ADC values on the FSE-PROPELLER DWI.

**Results:**

Benign tumors (ADC: 2.03 ± 0.31 × 10^-3^ mm^2^/s, T1: 1741.13 ± 662.64 ms, T2: 157.43 ± 72.23 ms) showed higher ADC, T1, and T2 values compared to malignant tumors (ADC: 1.46 ± 0.37 × 10^-3^ mm^2^/s, T1: 1390.06 ± 241.09 ms, T2: 97.64 ± 14.91 ms) (all P<0.05), while no differences were seen for PD values. ROC analysis showed that T2+ADC (cut-off value, > 0.55; AUC, 0.950) had optimal diagnostic performance vs. T1 (cut-off value, ≤ 1675.84 ms; AUC, 0.698), T2 (cut-off value, ≤ 113.24 ms; AUC, 0.855) and PD (cut off value, > 80.67 pu; AUC, 0.568) alone in differentiating malignant from benign lesions (all P<0.05); yet, the difference in AUC between ADC and T2+ADC or T2 did not reach statistical significance.

**Conclusion:**

Synthetic MRI and FSE-PROPELLER DWI can quantitatively differentiate malignant from benign HN tumors. T2 value is comparable to ADC value, and T2+ADC values could improve diagnostic efficacy., apparent diffusion coeffificient, head and neck tumors

## Highlights

- Synthetic MRI, FSE-PROPELLER DWI, and the combination of the two methods can all be used to quantitatively diagnose differential head and neck (HN) tumors.
- Synthetic MRI could constitute a new adjunct in diagnosing HN tumors.
- Synthetic MRI is comparable to FSE-PROPELLER DWI.
- The combined effect of the two methods was better than synthetic MRI used alone.

## 1 Introduction

Head and neck (HN) cancer is the 6^th^ most common cancer and the 9^th^ most common cause of cancer-related death (1, 2). Surgery is the most effective treatment for managing primary HN cancer. Yet, many patients present with advanced-stage tumors at the time of diagnosis and thus require more invasive treatment, including radiochemotherapy, immunotherapy, and targeted therapy (3). Also, diagnosis remains challenging considering its specific location (masses originating from the larynx, the nasopharynx, oropharynx, oral cavity hypopharynx, salivary glands, etc.). In addition, HN cancer might cause various symptoms that commonly accompany benign conditions (1). Thus, the differentiation of benign from cancerous masses is very important.

Tissue biopsy and pathologic examination remain the gold standard for evaluating the nature of HN lesions; nevertheless, only a part of the tissue can be obtained using this method. In addition, this approach is invasive and not always well accepted by the patient (4).

Endoscopy, head MRI, computed tomography (CT of the sinuses and head, dental cone beam CT), panoramic dental x-ray, and positron emission tomography (PET)/CT or chest imaging are the most common imaging methods used to assess the HN region. MRI is frequently used to detect, differentiate, grade, or draw the extent of HN tumors (1, 5). Among different MRI models, diffusion-weighted imaging (DWI) can quantitatively evaluate the Brownian motion diffusion of water molecules in tissues at a cellular level expressed as an apparent diffusion coefficient (ADC). DWI with a single-shot echo-planar sequence (SS-EP-DWI), which is commonly applied to investigate HN regions (6), is sensitive to chemical shifts, signal loss and geometric distortion, metallic dental implant-related magnetic susceptibility artifacts, and motion artifacts (7). Moreover, stimulus and fast spin echo DWI with periodically rotated overlapping parallel lines with enhanced reconstruction (FSE-PROPELLER DWI) is useful to distinguish parotid pleomorphic adenoma from Warthin tumor with less distortion of tumors than SS-EP-DWI (7). However, the value of FSE-PROPELLER DWI in distinguishing malignant from benign HN tumors has not been fully explored.

The major limitations of DWI include low signal-to-noise ratios and prolonged acquisition time. Over the years, a new synthetic MRI sequence based on a quantitative approach has been developed. This tool can estimate absolute physical properties, proton density (PD), and longitudinal and transverse relaxation times (T1, T2), which are independent of the MRI scanners or scanning parameters at a given field strength (8). Also, quantitative values (PD, T1, and T2) can be simultaneously acquired on the synthetic MRI, which enables a significant reduction in examination time with good accuracy and reproducibility for use in clinical practice (individual patient follow-up and comparison analysis (9–11). This approach has been used in the study of multiple systemic diseases of the brain (12), knee (13), spine (14), prostate (15), breast (16), bladder (17), and nasopharynx (18).

In this study, we further assessed the value of synthetic MRI in differentiating malignant from benign HN tumors compared with FSE-PROPELLER DWI and a combination of these two methods. To the best of our knowledge, this is the first study that focused on synthetic MRI and FSE-PROPELLER DWI to characterize HN tumors.

## 2 Materials and methods

### 2.1 Patients

MRI data from 48 consecutive patients (mean age ± standard deviation [SD], 48.08 years ± 15.01 [range, 18–76 years]) with HN tumors who were treated at our hospital between August 2022 and October 2022 were collected. The inclusion criteria were: (1) no tumor treatments before MR examinations; (2) all pathological examinations of samples were obtained by surgical resection or biopsy of the tumor; (3) synthetic MRI and FSE-PROPELLER DWI were acquired before surgical resection and biopsy; (4) the maximum tumor diameter was ≥ 6 millimeters. The exclusion criteria were: (1) MR images with obvious artifacts and poor quality; (2) patients previously treated. Subjects were divided into benign and malignant groups.

This study was approved by our institutional review board. Informed consent was waived.

### 2.2 Data collection

Demographic data included gender and age. All MRI acquisitions were performed on a 3T MR scanner (Premier, GE Healthcare, Milwaukee, WI, USA) in a supine position with a 21-channel head-neck coil. The following sequences were acquired in this study: axial T1-weighted image (T1WI), T2-weighted image (T2WI), synthetic MRI, and FSE-PROPELLER DWI with two b-values (0 and 800s/mm^2^). Detailed acquisition parameters are listed in **Table 1**.

**Table 1:** MRI Sequence Parameters.

Parameters	T1WI	T2WI	MAGIC	DWI
Imaging technique	FSE	Flex	Synthetic MRI	FSE-PROPELLER
Orientation	Axial	Axial	Axial	Axial
TR (ms)	693	3339	4000	3620
TE (ms)	6.7	80	13.3	50
Field of view (mm^2^)	220×220	220×220	220×220	220×220
Slice thickness (mm)	4	4	4	4
No. of slices	24	24	24	24
Nex	1	2	1	4
Fat suppression	NA	Dixon	NA	Fat sat
Acquisition matrix	320×224	280×224	224×224	120×50
Flip angle (degree)	111	110	NA	110
Acquisition time	40s	2 min 27s	3 min 38 s	3 min 20 s

T1WI, T1-weighted imaging; T2WI, T2 weighted imaging; DWI, diffusion-weighted imaging; TSE, turbo spin-echo; MAGIC, magnetic resonance image compilation; PROPELLER, periodically rotated overlapping parallel lines; TR, repetition time; TE, echo time; NA, not applicable.

### 2.3 Image analysis

Acquired data from synthetic MRI sequences were analyzed using magnetic resonance image compilation (MAGIC) software. Then, quantitative T1, T2, and PD maps were created and used for measurements to yield synthetic images and match the conventional images (19). The two radiologists (with 10 and 8 years of experience in head and neck MR imaging independently analyzed MR images) who were blind to the grouping manually drew the regions of interest (ROIs) on synthetic T2WI to obtain the PD, T1, and T2 values. Postprocessing of FSE-PROPELLER DWI was performed using the ADW 4.7 workstation (GE Healthcare). The axial routine MR images and DWI were used as references. ROIs were drawn on synthetic T2WI and ADC maps with care by avoiding necrosis, cystic degeneration, and bleeding areas at the slice with the largest tumor diameter and directly colocalized on the T1, T2, and PD maps. The size of ROIs was >25 mm^2^. Two radiologists measured three times. The average value was obtained by both radiologists in the analysis. Additionally, the largest lesion was selected for analysis if more than one HN lesion were present.

### 2.4 Statistical analysis

Shapiro-Wilk test was used to assess normality, while Levene’s test was used for variance homogeneity. The normally distributed variables were expressed as the means ± SD. Non-normally distributed variables were expressed as medians (interquartile ranges, IQRs). Differences in sex between the two groups were compared using a chi-square test. An independent samples t-test was used to compare the discrepancy in age between the two groups. The intraclass correlation coefficient (ICC) was used to assess the intraobserver agreement for quantitative parameters (19): value <0.40, 0.41-0.59, 0.60-0.74, and ≥0.75 indicated poor, fair, good, and excellent consistency, respectively. Pearson’s correlation coefficient was used to evaluate the correlation among parameters. The receiver operating characteristic (ROC) curve was conducted, and the area under the curve (AUC), sensitivity, specificity, negative predictive value (NPV), and positive predictive value (PPV) were further calculated to ascertain the diagnostic performance of quantitative parameters for differentiating malignant from the benign HN tumors. The diagnostic value of the combined ADC and T2 values (T2+ADC) was based on the logistic regression analysis. The method developed by DeLong et al. (20) was applied to compare AUCs. Statistical analysis was performed using MedCalc statistical software (version 19.6, MedCalc) and SPSS software (version 17.0, Chicago, IL, USA). P < 0.05 was considered statistically significant.

## 3 Results

### 3.1 General data

**Figure 1:**
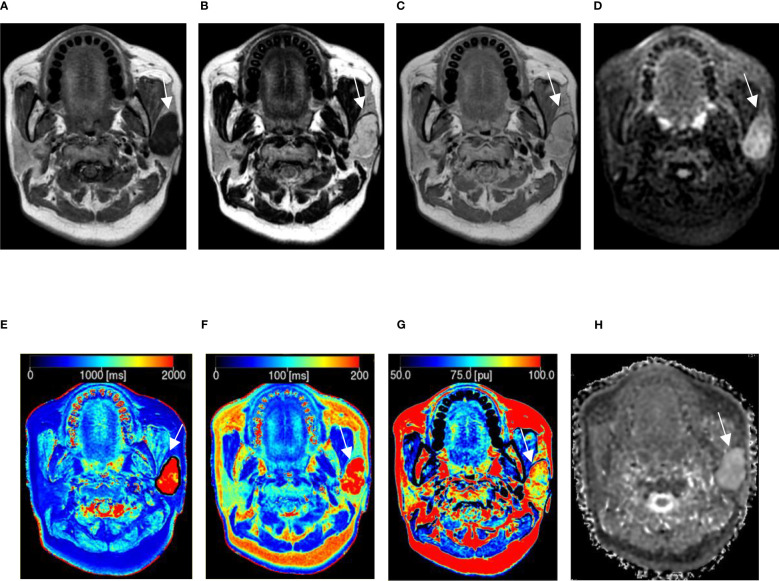
A 54-year-old woman with pleomorphic adenoma in the left parotid gland. **(A–C)** Proton density (PD), T1, and T2 images obtained from synthetic MRI. **(D)** Stimulus and fast spin echo diffusion-weighted imaging with periodically rotated overlapping parallel lines with enhanced reconstruction (b = 800 s/mm^2^). **(E–G)** Synthetic MRI-derived indicate that the mean T1, T2, and PD values measured by the two radiologists were 2227.50 ms, 265.17 ms, and 89.15 pu, respectively. **(H)** ADC map indicates that the mean apparent diffusion coefficient (ADC) value measured by the two radiologists was 2.40 × 10^-3^ mm^2^/s.

**Figure 2:**
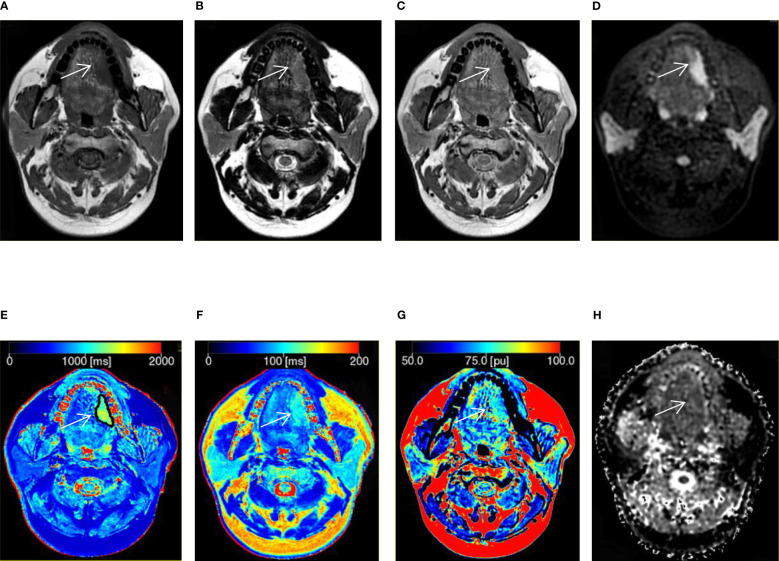
A 41-year-old man with squamous cell carcinoma in the tongue. **(A–C)** T1, T2, and proton density (PD) weighted images obtained from synthetic MRI. **(D)** Stimulus and fast spin echo diffusion-weighted imaging with periodically rotated overlapping parallel lines with enhanced reconstruction (b = 800 s/mm^2^). **(E–G)** Synthetic MRI-derived maps indicate that the mean T1, T2, and PD values measured by the two radiologists were 1214.67 ms, 105.17 ms, and 80.39 pu, respectively. **(H)** ADC map indicates that the mean apparent diffusion coefficient (ADC) value measured by the two radiologists was 1.52 × 10^-3^ mm^2^/s.

A total of 48 patients with histologically diagnosed HN tumors were assessed. Demographics are listed in **Tables 2** and **3**. In addition, representative images of benign and malignant tumors are depicted in **Figures 1** and **2**. There was no difference in age (t = -1.392, P > 0.05) and gender between the two groups (χ^2^ = 0.689, P = 0.406).

**Table 2:** Histologic types and locations of head and neck tumors.

Benign/malignant	Histologic types	Locations	Gender(M/F)	Age (y)	T1(ms)	T2(ms)	PD(pu)	ADC(×10^-3^ mm^2^/s)
Benign	Pleomorphic adenoma	Parotid gland	M	19	1854	147.17	89.4	2.15
Benign	Pleomorphic adenoma	Parotid gland	M	50	1230.67	117.83	68.74	1.67
Benign	Pleomorphic adenoma	Parotid gland	F	48	2991	317.33	90.99	2.59
Benign	Pleomorphic adenoma	Submandibular gland	F	54	1837	152.33	84.97	2.15
Benign	Pleomorphic adenoma	Parotid gland	F	49	1334.5	110.83	80.67	1.86
Benign	Pleomorphic adenoma	Parotid gland	F	32	916.5	114.17	86.25	1.76
Benign	Pleomorphic adenoma	Parotid gland	M	35	1292.5	99.17	74.17	1.84
Benign	Pleomorphic adenoma	Parotid gland	F	24	1712.84	143	86.15	2.03
Benign	Pleomorphic adenoma	Parapharyngeal space	M	59	1287.33	153.5	80.47	1.95
Benign	Pleomorphic adenoma	Parotid gland	F	63	1854.67	174.17	87.07	2.18
Benign	Pleomorphic adenoma	Parotid gland	M	52	1573.5	140.5	88.60	1.94
Benign	Pleomorphic adenoma	Parotid gland	F	32	3332.5	304.5	97.52	2.42
Benign	Pleomorphic adenoma	Parotid gland	M	21	2810.34	274.34	98.15	2.58
Benign	Pleomorphic adenoma	Parotid gland	F	42	1548.84	118.17	86.09	1.86
Benign	Pleomorphic adenoma	Parotid gland	F	54	2227.5	265.17	89.15	2.40
Benign	Basal cell adenoma	Parotid gland	M	53	1382.33	93.17	80.3	1.54
Benign	Basal cell adenoma	Parotid gland	F	57	1431	96.5	77.84	1.79
Benign	Basal cell adenoma	Parotid gland	M	44	1401.83	97.5	79.19	1.71
Benign	Basal cell adenoma	Parotid gland	M	62	817	120.67	89.25	2.34
Benign	Basal cell adenoma	Parotid gland	F	41	1986.84	108.5	87.99	1.90
Malignant	Squamous cell carcinoma	Tongue	M	54	1329.5	98.84	83.22	1.55
Malignant	Squamous cell carcinoma	Hypopharynx	M	64	1166.17	78.33	89.83	1.72
Malignant	Squamous cell carcinoma	Tongue	M	32	1850	93.5	90.17	1.84
Malignant	Squamous cell carcinoma	Nasopharynx	M	18	1422.67	100	85.38	1.04
Malignant	Squamous cell carcinoma	Tongue	F	62	1143.67	86.67	80.5	2.49
Malignant	Squamous cell carcinoma	Nasopharynx	M	56	1326.17	95.67	85.85	1.15
Malignant	Squamous cell carcinoma	Tongue	M	53	1244.34	91.84	82.6	1.54
Malignant	Squamous cell carcinoma	Nasopharynx	F	36	1175.34	80.17	87.25	1.34
Malignant	Squamous cell carcinoma	Vocal cords	M	52	2067.17	88.67	90.14	2.41
Malignant	Squamous cell carcinoma	Nasopharynx	M	37	1197.17	69.33	81.54	1.56
Malignant	Squamous cell carcinoma	Tongue	F	48	1334.17	101.5	85.83	1.44
Malignant	Squamous cell carcinoma	Nasopharynx	M	27	1054.5	82.17	80.92	1.07
Malignant	Squamous cell carcinoma	Nasopharynx	M	59	1214.17	93.5	89.35	1.14
Malignant	Squamous cell carcinoma	Parotid gland	M	76	1471.17	113.34	82.87	1.50
Malignant	Squamous cell carcinoma	Buccal mucosa	F	47	1348.84	102.4	87.78	1.43
Malignant	Squamous cell carcinoma	Tongue	M	41	1214.67	105.17	80.39	1.52
Malignant	Squamous cell carcinoma	Buccal mucosa	F	65	1276.84	92.17	79.27	1.60
Malignant	Squamous cell carcinoma	Tongue	M	69	1283.67	78.83	84.54	1.43
Malignant	Lymphoma	Parapharyngeal space	F	49	1308.5	83.83	83	0.98
Malignant	Lymphoma	Nasopharynx	M	66	1398	89.67	84.97	1.03
Malignant	Lymphoma	Parotid gland	F	52	1483	125.33	82.25	1.54
Malignant	Lymphoma	Submandibular gland	F	73	1571.84	109.34	87.9	1.24
Malignant	Lymphoma	Tonsil	F	50	1649	110.84	89.48	1.16
Malignant	Acinar cell carcinoma	Parotid gland	F	58	1675.84	121.17	86.32	1.77
Malignant	Acinar cell carcinoma	Parotid gland	M	32	1181	103.33	89.43	1.58
Malignant	Rhabdomyosarcoma	Parotid gland and neck	F	20	1833.67	133.84	83.92	1.31
Malignant	Melanoma	Paranasal sinus	M	74	1239.5	99.84	89.54	1.17
Malignant	Plasmacytoma	Parapharyngeal space	F	47	1461.17	104.5	85.95	1.20

**Table 3:** Demographics and the parameters of patients with head and neck tumors.

Group	Gender (M/F)	Age(y)	T1 (ms)	T2 (ms)	PD (pu)	ADC (×10^-3^ mm^2^/s)
Benign tumor	9/11	44.55 ± 13.44	1741.13 ± 662.64	157.43 ± 72.23	85.15 ± 7.17	2.03 ± 0.31
Malignant tumor	16/12	50.61 ± 15.80	1390.06 ± 241.09	97.64 ± 14.91	85.36 ± 3.37	1.46 ± 0.37
χ^2^*/*t*/*t’	0.689^a^	-1.392^b^	2.265^c^	3.647^c^	-0.125^c^	5.762^c^
*P* value	0.406	0.171	0.033	0.002	0.901	<0.001

Unless otherwise indicated, data are mean ± standard deviation (SD). ADC, apparent diffusion coefficient; PD, proton density, ^a^ Data is χ^2^, ^b^ Data is t,^c^ Data is t’.

### 3.2 Interobserver reliability

ICC analyses showed excellent consistency in the ADC, T1, T2, and PD values assessed by the two physicians: the ICC values were 0.976 (95% CI 0.958 - 0.987, P = 0.000), 0.936 (95% CI 0.988 - 0.997, P = 0.000), 0.996 (95% CI 0.993 - 0.998, P 0.001), and 0.823 (95% CI 0.706 - 0.897, P = 0.000), respectively.

### 3.3 Correlation analysis

Pearson’s correlation analysis showed a significant positive correlation between the T1 and T2 values (r = 0.854, P < 0.001), T1 and PD values (r = 0.574, P < 0.001), T1 and ADC values (r = 0.565, P < 0.001), T2 and PD values (r = 0.495, P < 0.001), and T2 and ADC values (r = 0.646, P< 0.001), respectively. There was no significant positive correlation between PD and ADC values (r = 0.281, P = 0.053).

### 3.4 MRI values between the two groups

The T1 value (1741.13 ± 662.64ms), T2 value (157.43 ± 72.23ms), and ADC value (2.03 ± 0.31 × 10^-3^ mm^2^/s) of the benign group was higher compared to the malignant group (T1: 1390.06 ± 241.09ms, t’ = 2.265, P = 0.033; T2: 97.64 ± 14.91ms, t’ = 3.647, P = 0.002; ADC: 1.46 ± 0.37 × 10^-3^ mm^2^/s, t' = 5.762, P < 0.001). Yet, no significant differences were found in PD values between the two groups (t’ = -0.125, P = 0.901).

### 3.5 Comparison of ROC curves

The AUC, cut-off, sensitivity, specificity, PPV, and NPV of each parameter discriminating malignant from benign lesions are summarized in **Table 4**.

**Table 4:** Diagnostic performance of MRI values and combined values for differentiating malignant from benign lesions.

Variable	Cut off	AUC (95%CI)	Sensitivity (%)	Specificity (%)	PPV (%)	NPV (%)	*P value
T1 (ms)	1675.84	0.698(0.549-0.822)	89	45	69	75	0.0161
T2 (ms)	113.34	0.855(0.546-0.801)	89	70	81	82	<0.0001
PD (pu)	80.67	0.568(0.429-0.700)	89	35	66	70	0.408
ADC (×10^−3^ mm^2^/s)	1.60	0.906(0.787-0.971)	82	95	96	79	<0.0001
T2+ADC	0.55	0.950(0.845-0.992)	89	90	93	86	<0.0001

ADC, apparent diffusion coefficient; PD, proton density; AUC, area under the curve; NPV, negative predictive value; PPV, positive predictive value, *p values are for the differences between benign and malignant head and neck tumors.

**Figure 3:**
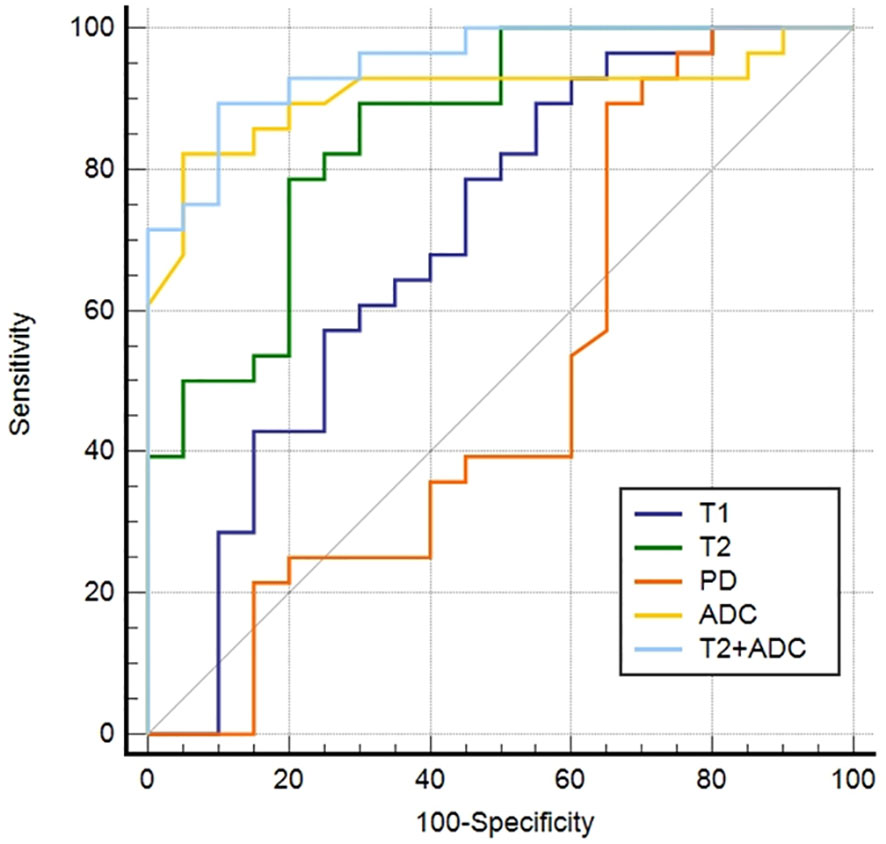
Receiver operating characteristic curves of the parameters derived from synthetic MRI and fast spin echo diffusion-weighted imaging with periodically rotated overlapping parallel lines with enhanced reconstruction in differentiating malignant from the benign head and neck tumors.

ROC curves for differentiating malignant from benign lesions are depicted in **Figure 3**. T2+ADC (cut-off value, > 0.55; AUC, 0.950) showed optimal diagnostic performance, which was better than that of T1 (cut-off value, ≤ 1675.84 ms; AUC, 0.698), T2 (cut-off value, ≤ 113.24 ms; AUC, 0.855) and PD (cut off value, > 80.67 pu; AUC, 0.568) (P = 0.0030, 0.0464, and P < 0.0001, respectively). The diagnostic performance of ADC was better than T1 and PD (P = 0.0138 and 0.0005, respectively), but the difference in AUC between ADC (cut-off value, ≤ 1.60 × 10^-3^ mm^2^/s; AUC, 0.906) and T2+ADC or T2 did not reach significance (P = 0.2648 and 0.4604, respectively). The diagnostic performance of T2 was better than PD (P = 0.0075); however, the difference in AUC between T2 and T1 did not reach statistical significance (P = 0.0549).

## 4 Discussion

We examined the tissue magnetic property parameters acquired on the synthetic MRI with MAGIC and ADC acquired on the FSE-PROPELLER DWI in discriminating malignant from benign HN lesions. The purpose of evaluating parameter correlation is to identify preferred parameters and facilitate the translation of scientific research into clinical practice. Except for PD values, T1, T2, and ADC values were lower in malignant than in benign HN tumors. ADC, T1, and T2 values are widely used parameters for differentiating malignant from benign HN tumors. The diagnostic performance of the T2 value is comparable to the ADC value. However, the diagnostic performance of the T1 and PD values was not as good as that of the ADC value. We also found that T2+ADC showed optimal diagnostic performance.

In this study, the malignant tumor had a lower T1 value related to hyper-cellularity, smaller extracellular space, and lower free water content (18). Meng et al. found that the T1 value for nasopharyngeal carcinoma was significantly lower than that for benign hyperplasia in the nasopharynx, regardless of the ROI used (18). Contrary Gao and his team found no difference in the T1 values between malignant and benign breast lesions (21). The different types of tumors in these studies may contribute to the discrepancy.

T2 value can be affected by various factors, including the main magnetic field strength and the intrinsic properties of the tissue and the environment. Tissue water content is the most important influencing factor (22). A previous study reported a linear relationship between the T2 value and water content (23); thus, increased T2 values indicate increased tissue water content (24). In our study, 75% of benign HN tumors were pleomorphic adenomas followed by basal cell adenomas, and 64% of malignant HN tumors were squamous cell carcinomas followed by lymphomas. We speculate that the higher T2 values of benign HN tumors could be ascribed to their tissue composition, lower cell density, and higher free water content (5). Nevertheless, the lower T2 values in malignant tumors are due to the increased solid components, smaller extracellular spaces, and lower free water content (5, 18, 25). Several studies have reported higher T2 values in benign breast lesions vs. in malignant lesions (21). This difference could be attributed to increased tissue water content or interaction between water and alkaline metal cations in the pathological tissue (22).

PD value, which primarily reflects the water content in tissue, is frequently used in brain imaging (26). This study found that the difference in PD value in the malignant compared with benign HN tumors was not statistically significant. Yet, Gao et al. demonstrated that the PD value was significantly lower in malignant than that benign breast lesions (21). The different types of tumors enrolled in these studies may contribute to the contradiction.

Here we found that the ADC values of malignant tumors were significantly lower than those of benign tumors. Higher ADC values correlate with lower cellularity (27). Malignant tumors demonstrate lower ADC values than benign tumors due to their relatively higher cellularity (4). Srinivasan et al. also found that malignant lesions showed lower ADC values than benign lesions (28).

In this study, the overall diagnostic performance of synthetic MRI-derived parameters in discriminating malignant from the benign HN lesions was inferior to the ADC value. However, the diagnostic performance showed no significant differences between ADC and T2 values. Also, T2+ADC showed optimal diagnostic efficacy in distinguishing malignant from benign tumors; T2+ADC showed a significantly higher differential performance vs. T1, T2, or PD value alone, but it did not improve the diagnostic performance of the ADC value. Despite this, the PD, T1, and T2 are intrinsic magnetic properties and independent from the MRI scanners or scanning parameters at a given field strength (15), predicting the potential advantage of using synthetic MRI-derived parameters compared to the ADC value alone (17). In addition, synthetic MRI can generate multiple contrast-weighted images and quantification maps in a single scan, greatly improving work efficiency (10). Thus, synthetic MRI plus FSE-PROPELLER DWI might be a promising tool for differentiating benign from malignant HN lesions.

The present study has a few limitations. First, this is a single-center retrospective study with small sample size, next, we will expand the sample size for further study. Second, ROIs were manually drawn at the slice with the largest tumor diameter, leading to potential operator errors. In the future, the whole tumor should be selected to determine whether the tumor volume is more meaningful and accurate for tumor characterizing. Finally, test-retest repeatability was not assessed.

## 5 Conclusion

The quantitative T1, T2, and PD values obtained by synthetic MRI and ADC value obtained by FSE-PROPELLER DWI helped discriminate malignant from benign HN tumors. The overall diagnostic performance of synthetic MRI was inferior to FSE-PROPELLER DWI. However, the T2 value was comparable to the ADC value, and the combination of synthetic MRI and FSE-PROPELLER DWI could provide improved diagnostic efficacy.

## Data availability statement

The raw data supporting the conclusions of this article will be made available by the authors, without undue reservation.

## Author contributions

BW: Conceptualization, Methodology, Software, Data curation, Writing - original draft, Visualization, Investigation, Writing - review & editing. ZZ: Data curation, Writing - original draft, Visualization, Investigation, Writing - review & editing. JZ: data analysis, statistical analysis. LL: Data curation. ZL: Data curation, Writing - original draft. XM: Visualization, Investigation. KW: Conceptualization, Methodology, Software, Writing - review & editing. LX: Conceptualization, Methodology, Software, Validation, Writing - review & editing. YZ: Supervision. JC: Supervision. All authors contributed to the article and approved the submitted version.
